# A very picky eater: Species‐level prey selection in the endangered Rhone streber [ *Zingel asper* (L. 1758)]

**DOI:** 10.1111/jfb.70083

**Published:** 2025-05-26

**Authors:** Kurt Villsen, Emmanuel Corse, Gaït Archambaud‐Suard, Vincent Dubut

**Affiliations:** ^1^ Aix Marseille Univ Avignon Université, CNRS, IRD, IMBE Marseille France; ^2^ INRAE, UR RiverLy Villeurbanne France; ^3^ Centre Universitaire de Formation et de Recherche de Mayotte Dembeni France; ^4^ MARBEC, University of Montpellier, CNRS, Ifremer, IRD Montpellier France; ^5^ INRAE, Aix Marseille Univ, RECOVER Aix‐en‐Provence France; ^6^ ADENEKO Saint‐Girons France

**Keywords:** conservation, metabarcoding, prey selection, trophic ecology

## Abstract

Prey preferences are important drivers of predator–prey interactions and trophic network structure. We present a species‐level selection analysis for the endangered *Zingel asper* (L. 1758) and its prey within the *Baetis* (Ephemeroptera) genus. By combining robust diet metabarcoding data with fine‐scale prey community data, we revealed that *Z. asper* selected four *Baetis* species differently, despite being largely considered as ecologically similar species. Our results suggest that fine‐resolution analysis of prey selection may be key for understanding trophic interactions and for improving the conservation and management of fish.

Trophic interactions are fundamental to evolutionary and ecological processes (Hanley & La Pierre, 2015). In particular, prey preferences constitute a critical component of every organism's feeding ecology. According to Optimal Foraging Theory, predators are expected to prefer prey that maximise energy gain relative to the energy required to obtain them, with preferred prey representing optimal prey (MacArthur & Pianka, 1966; Schoener, 1971). The Optimal Foraging Theory also predicts that when high‐value prey become scarce, predators will broaden their diet (Ivlev, 1961; Perry and Pianka, 1997). Consistent with this prediction, preferred prey availability has been shown to drive predator foraging strategies (Tinker et al., 2008; Villsen et al., 2024). Furthermore, selective foraging greatly modifies trophic interactions (i.e., predator–prey and competitive) and contributes to community structure and dynamics (Abrams, 2000; Allan, 1983). A detailed understanding of prey preferences is therefore essential to characterise a predator's role within the food web (e.g., Ludwig et al., 2024; Siegenthaler et al., 2019).

Predator preferences underly predator–prey interactions, as preferred prey are disproportionately consumed based on their environmental availability (Chesson, 1983; Sih et al., 1985). Accordingly, prey preferences are assessed by comparing prey availability in the environment with predators' diets (Chesson, 1983). However, selection analyses are often performed at broad taxonomic levels (i.e., genus, family or higher), which inherently leads to the homogenisation of prey traits (e.g., Cochran‐Biederman & Vondracek, 2017; Newkirk & Schoenebeck, 2018). High‐resolution prey preference analysis (i.e., to the species level) has recently been shown to greatly improve our understanding of predator–prey interactions in food webs (Clare et al., 2019; Cuff et al., 2022). Species‐level prey preference analysis could therefore provide essential insights to inform conservation and management strategies (Naman et al., 2022).

The Rhone streber [*Zingel asper* (L. 1758)] is an endangered invertivorous benthic fish that is endemic to the Rhône River basin (Ford, 2024). *Z. asper* inhabits medium‐to‐small rivers with rocky substrates (Labonne et al., 2003), and individuals exhibit limited diel displacement ranges (50–200 m; Danancher et al., 2004). The diet of *Z. asper* is dominated by *Baetis fuscatus* (Ephemeroptera), which constitutes ~45% of its total diet (Villsen, Corse, Meglécz, et al., 2022). However, several other *Baetis* species (*Baetis scambus*, *Baetis rhodani, Baetis buceratus and Baetis lutheri*) also occur in the diet of *Z. asper* (Villsen, Corse, Meglécz, et al., 2022). To assess species‐level selectivity in *Z. asper* among *Baetis* species, we re‐examined previously acquired macroinvertebrate data to obtain species‐level information on prey abundance and conducted prey selection analysis.

## 1 DATASET DESCRIPTION AND STATISTICAL ANALYSES

**FIGURE 1:**
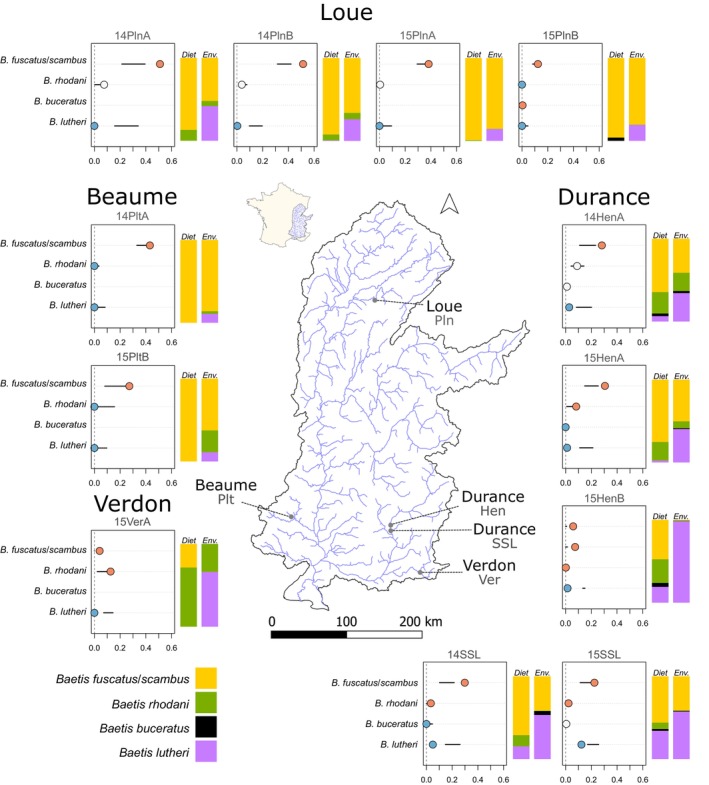
*Zingel asper* prey preferences (boxes) and composition of *Baetis* species in the diet and in the environment (bar plots). In selection graphs, the position of dots along the *x*‐axis indicates observed dietary proportions; the colour of dots indicates deviations from expected frequencies of trophic interactions: red, higher than expected (consumed more frequently than expected); blue, lower consumption than expected; white, as expected (in proportion to relative abundance). Horizontal lines denote 95% confidence limits of null model expectations of predation. Bar plots indicate the proportion of *Baetis* species' relative abundance in the diet (*Diet*) and the environment (*Env*.). [Correction added on 23 June 2025, after first online publication: Figure 1 has been corrected in this version.]

This study uses metabarcoding diet data and macroinvertebrate data from Villsen, Corse, Archambaud‐Suard, et al. (2022), Villsen, Corse, Meglécz, et al. (2022) and Villsen et al. (2024). The dataset covers the four extant *Z. asper* populations, which are located in the Durance, Verdon, Beaume and Loue rivers (Figure 1; Table 1). Metabarcoding data were obtained from *Z. asper* faeces using a robust and benchmarked procedure (Corse et al., 2017, 2019; González et al., 2023). Although metabarcoding provides fine‐scale dietary data for reconstructing food webs (Clare et al., 2019; Cuff et al., 2022), it is often criticised for being poorly quantitative (Lamb et al., 2019). This issue was addressed using the minimum number of individuals (MNI; White, 1953), which is a quantitative metric based on the number of distinct haplotypes found for a given prey species in each faeces (Corse et al., 2017; Villsen, Corse, Meglécz, et al., 2022).

**TABLE 1:** Diet and macroinvertebrate sampling information.

River	Sampling site	Geographical co‐ordinates	Campaign ID	Season	Campaign date	Diet samples	Benthos samples	*Baetis* abundance (unidentified)
Durance	Hen	5°55′29″ E 44°18′46″ N	14HenA	Spring	14 May	33^a^	90^b^	1437 (0)
			15HenA	Spring	15 May	30^a^	90^b^	1766 (133)
			15HenB	Autumn	15 November	27^a^	90^c^	2473 (5)
Durance	SSL	5°55′17″ E 44°14′50″ N	14SSL	Summer	14 August	25^b^	45^b^	2300 (25)
			15SSL	Summer	15 September	44^c^	60^c^	1260 (118)
Verdon	Ver	6°20′58″ E 43°44′15″ N	15VerA	Summer	15 July	20^a^	61^b^	203 (8)
Beaume	Plt	4°16′39″ E 44°27′18″ N	14PltA	Spring	14 June	35^a^	90^b^	3368 (74)
			15PltB	Autumn	15 October	30^a^	90^b^	169 (6)
Loue	Pln	5°49′36″ E 47°0′4″ N	14PlnA	Spring	14 June	21^a^	90^b^	2137 (30)
			14PlnB	Summer	14 September	49^a^	90^b^	2269 (34)
			15PlnA	Spring	15 July	41^a^	90^b^	1378 (12)
			15PlnB	Autumn	15 September	48^a^	90^b^	600 (27)

*Note*: Metabarcoding diet data and macroinvertebrate data were obtained from.

^a^ Villsen, Corse, Meglécz, et al. (2022).

^b^ Villsen et al. (2024).

^c^ Villsen, Corse, Archambaud‐Suard, et al. (2022).

Macroinvertebrate abundance was estimated from 45 to 90 Surber samples (0.05 m^2^) per sampling campaign (see Supplementary Materials for additional details). To test for within‐genus *Baetis* selectivity in *Z. asper*, we focused on 12 campaigns from Villsen, Corse, Archambaud‐Suard, et al. (2022); Villsen et al. (2024) for which species‐level identification of *Baetis* specimens was available (Table 1). However, *B. fuscatus* (the main prey species of *Z. asper*) is not morphologically distinguishable from *B. scambus* (a secondary prey species) (Elliott & Humpesch, 2010). We therefore grouped *B. fuscatus* and *B. scambus* together (hereafter referred as *B. fuscatus/scambus*) for conducting selection analyses. Most *Baetis* specimens (minimum 90% per sampling campaign) could be assigned to *B. fuscatus/scambus*, *B. rhodani*, *B. buceratus* or *B. lutheri* (Table 1). In certain cases, however, species‐level taxonomic assignment was not feasible (e.g., for small individuals; <2 mm). In this case, unidentified individuals were assigned to *B. fuscatus/scambus, B. rhodani, B. buceratus* and *B. lutheri* based on the relative proportions of identified individuals, for each sampling site–date combination, separately.

Prey selection analyses were performed by comparing observed macroinvertebrate proportions in *Z. asper* faeces to their proportion in the environment. Only macroinvertebrate taxa found in ≥5% of *Z. asper* diets were included (Tables S1 and S2). The proportion of macroinvertebrates measured in the river was based on count data, whereas the proportion of macroinvertebrates in faeces was based on MNI. We excluded diet data for young‐of‐the‐year individuals due to their distinct diet compared to older fishes (see: Villsen, Corse, Meglécz, et al., 2022). Selection analyses were performed separately for each sampling campaign using the *econullnetr* package (*generate_null_net*, sims = 1000). The selection analysis relies on null models that estimate expected prey consumption based on observed individual diet breadth and prey environmental availability (Vaughan et al., 2018). Three outcomes are possible for selection tests: (i) positive selection [observed consumption > null expectation 95% confidence interval (CI)], (ii) negative selection (observed < null 95% CI) and (iii) neutral selection (observed = null 95% CI).

## 2 ZINGEL ASPER PREY PREFERENCES WITHIN THE *BAETIS* GENUS

Our results highlight strongly contrasting prey preferences within the *Baetis* genus for *Z. asper*. *Z. asper* exhibited a strong and consistent preference for *B. fuscatus/scambus*, with only minor variation across sites and seasons (Figure 1). In contrast, the second most abundant *Baetis* species, *B. lutheri*, was consistently negatively selected by *Z. asper* (Figure 1). As for *B. rhodani*, although it was neutrally or slightly negatively selected in Beaume and Loue rivers, it was positively selected in the Durance and Verdon rivers (five out of six sampling campaigns). This was most notable in the Verdon River where the diet of *Z. asper* was dominated by *B. rhodani* (Figure 1). Lastly, *B. buceratus* was both the rarest *Baetis* species in the environment and the least consumed by *Z. asper*.

Although our species‐level analyses revealed contrasting patterns of prey selection, genus‐level analyses showed positive selection for *Baetis* (Figure S1). Our results demonstrate that positive genus‐level selection is largely driven by positive selection for *B. fuscatus/scambus* (and *B. rhodani* to a lesser extent), whereas *B. lutheri* was systematically negatively selected. Furthermore, as *B. scambus* rarely appeared in the diet of *Z. asper* (Table S3), we assume that the selection results obtained for *B. fuscatus/scambus* mainly reflect preference for *B. fuscatus*.

## 3 THE IMPLICATIONS OF SPECIES‐LEVEL PREY SELECTION IN *Z. asper*

Aggregating prey at broad taxonomic levels (e.g., genus or family) can obscure species‐specific predator–prey interactions, especially when a predator preferentially interacts with a subset of species within a broader taxonomic grouping. This is best illustrated when comparing species‐level preferences to genus‐level preferences (Figure S1). Although the *Baetis* genus is consistently positively selected across all sites, we demonstrated that *Z. asper* almost exclusively interacted with a subset of the *Baetis* species (here, *B. fuscatus*, and to a lesser extent, *B. rhodani*). Our results therefore highlight the importance of resolving predator–prey interactions to the species level. Indeed, our results show that even supposedly ecologically similar species (such as *Baetis* spp.) can have distinct interactions with predators. As prey and predator traits interact to determine how predators forage and select their prey (O'Brien, 1979), our results suggest that intra‐*Baetis* trait variation drives the observed prey preferences. Predator traits (e.g., physiology, morphology, behaviour) predispose predators to prefer certain prey traits (e.g., abundance, morphology, palatability, habitat use, predator‐avoidance behaviour), leading to the disproportionate consumption of certain prey over others (O'Brien, 1979; Worischka et al., 2012, 2015). Consequently, as prey preferences are expected to reflect optimal foraging choices (Pyke et al., 1977), it follows that *B. fuscatus* exhibits traits that may allow *Z. asper* to maximise its energy gain. For example, many predators exhibit density‐dependent selection, choosing to specialise on highly abundant prey to maximise energy intake (Murdoch, 1969; Schoener, 1971; Worischka et al., 2015). During the main growing season of *Z. asper* (spring and summer; Cavalli et al., 2003; Monnet et al., 2022), *B. fuscatus* was the most abundant *Baetis* species, which may partially explain the observed pattern of prey selection: *Z. asper* may specialise on the most abundant prey in summer and spring. However, although *Z. asper* was able to supplement its diet with *B. rhodani*, *Z. asper* was effectively unable to shift to *B. lutheri* when *B. fuscatus* was scarce. Functional trait analyses have been proven to be powerful tools for understanding the mechanisms that drive prey preferences (e.g., Ludwig et al., 2024; Rodríguez‐Lozano et al., 2016; Worischka et al., 2015). For example, prey functional traits like habitat use, diel period or predator avoidance strategies may make it difficult to shift between exploiting the different species at any given time (Culp et al., 1991; Scrimgeour et al., 1994; Worischka et al., 2012). However, knowledge of the ecology of *Baetis* species remains largely limited to the genus level and assumes functional similarity across species (Bauernfeind & Soldan, 2012; Tachet et al., 2010). Our results challenge this assumption, suggesting that *B. lutheri* may differ in some unidentified functional traits that reduce its value (e.g., palatability) or accessibility (e.g., escape strategy, microhabitat preference) as prey. Indeed, if all *Baetis* species were equally beneficial to *Z. asper*, one would expect them to show similar patterns of interaction with this predator. Future studies that address the specific functional traits of *Baetis* species will therefore be necessary to understand the exact mechanisms that underly prey selection in *Z. asper*.

High‐quality prey availability directly affects life‐history traits like survival, growth and energy reserves (Elliott & Hurley, 2000; Garvey & Whiles, 2016). Therefore, conservation strategies for *Z. asper* that require estimates of habitat quality, including river management and reintroduction programmes, should account for the availability of preferred prey species. We previously demonstrated that preferred prey availability is a key driver of individual trophic trait variation in *Z. asper* (Villsen et al., 2024). In the present study, we highlight the importance of distinguishing between *Baetis* species to accurately characterise the prey preferences of *Z. asper*. Consequently, estimating the abundance of each *Baetis* species separately should be more informative than assessing total genus‐level abundance for the conservation and management of *Z. asper* populations. Especially, although *B. lutheri* can be quite abundant in the environment, it appears to be of little relevance to the population dynamics of *Z. asper*.

## 4 METHODOLOGICAL CONSIDERATIONS FOR FUTURE STUDIES

Diet metabarcoding has now proven to be a powerful tool for resolving complex interactions in trophic networks (e.g., Casey et al., 2019; Fablet et al., 2024), but it requires robust and standardised procedures to ensure the reliability of data (Calderón‐Sanou et al., 2020; Villsen, Corse, Meglécz, et al., 2022). Two key challenges of using metabarcoding in trophic ecology studies are (i) extracting quantitative information from metabarcoding data and (ii) standardising taxonomic resolution, both within and across datasets (Cuff et al., 2022). We addressed the first point using the MNI statistic to obtain a conservative quantification of prey consumption. Indeed, when coupled with thorough sampling (i.e., 20–48 faeces samples per campaign), we previously demonstrated that the MNI metric provides ecologically reliable estimates from metabarcoding derived data (Villsen, Corse, Archambaud‐Suard, et al., 2022; Villsen, Corse, Meglécz, et al., 2022; Villsen et al., 2024). To address the second point, expert knowledge of the subtle morphological differences between *Baetis* species was essential to standardise taxonomic resolution between metabarcoding diet data and environmental prey availability data.

Most studies assessing prey selectivity summarise predator preferences at the genus or even family level, thereby overlooking patterns of selectivity within prey genera (e.g., Cochran‐Biederman & Vondracek, 2017; Newkirk & Schoenebeck, 2018). Even when high‐resolution metabarcoding data are available, analyses of prey preferences often remain restricted to broader taxonomic levels (e.g., Siegenthaler et al., 2019). In this study, by combining high‐resolution diet metabarcoding data and high‐resolution morphological data, we demonstrated that *Z. asper* exhibits species‐specific prey preferences within the *Baetis* genus. However, identifying macroinvertebrates based on morphology is challenging, as it is time‐consuming and requires expert knowledge of taxonomy and morphology. To overcome this challenge, future studies could use metabarcoding to characterise both the diet and the prey community, ensuring consistent taxonomic resolution for both datasets (Elbrecht & Steinke, 2019; Macher et al., 2025).

Furthermore, prey selectivity analysis are highly sensitive to the sampling methodology used to estimate prey abundance (Cuff et al., 2024). In river ecosystems, estimating food availability for fish is very challenging (Ouellet et al., 2024). Benthic macroinvertebrate community sampling often targets specific habitat types (e.g., riffles, runs, pools) to estimate prey availability (e.g., Esnaola et al., 2021; Sánchez‐Hernández et al., 2021). However, dividing continuous habitat conditions (e.g., water velocity, depth, slope) into discrete categories likely overlooks important variation in prey and habitat conditions for benthic predators. The random sampling approach used in this study (Villsen et al., 2024; for a similar approach, see Heino et al., 2004) was designed to be representative of the overall macroinvertebrate community for each sampling campaign. Our sampling protocol was assumed to be appropriate for *Z. asper*, as individuals tend to space themselves out within populations rather than converging on preferred habitats (Labonne & Gaudin, 2005). Although this approach requires substantial sampling effort (45–90 Surber samples per sampling campaign), it accounts for the spatial heterogeneity of the prey community and provides reliable abundance estimates (Villsen et al., 2024). However, despite using a fine‐scale sampling method, we noted one case in the Verdon River wherein *B. fuscatus/scambus* was not detected, even though *B. fuscatus* appeared in the diet of *Z. asper* (Table S3). This illustrates how hard it can be to comprehensively measure prey availability, especially for low‐abundance prey species.

Overall, this study illustrates how diet data derived from short‐term metabarcoding data can be combined with fine‐scale snapshot estimates of prey abundance to reveal species‐level prey preferences. This high‐resolution analysis of prey preferences has deepened our understanding of the processes, shaping interactions between *Z. asper* and its prey, while also offering fine‐scale insights for the conservation and management of this endangered species.

## AUTHOR CONTRIBUTIONS

Vincent Dubut, Kurt Villsen and Emmanuel Corse conceived and designed the study. Gaït Archambaud‐Suard morphologically identified Baetis spp. specimens. Kurt Villsen performed statistical analyses. Kurt Villsen and Vincent Dubut wrote the original draft. Emmanuel Corse and Gaït Archambaud‐Suard contributed to further writing and editing.

## FUNDING INFORMATION

This study was funded by École Doctorale des Sciences de l'Environnement (ED251, Aix Marseille Université).

## Supporting information


**Figure S1.** *Zingel asper* prey preferences at genus‐ and family levels (above) and within the *Baetis* genus (below). The position of dots along the *x*‐axis indicates observed consumption (dietary proportions; 0–1); the colour of dots indicates deviation from expected frequencies of trophic interactions; blue, lower consumption than expected; white, as expected (in proportion to relative environmental abundance); red, higher than expected (consumed more frequently than expected). Horizontal lines denote 95% confidence limits of null model expectations of prey consumption. Genus‐ and family‐level tests of prey preferences were extracted from Villsen et al. (2024). Note that selection tests for Heptageniidae do not include *Epeorus* or *Rhithrogena*, and Chrionomidae does not include Orthocladiinae. The colour of campaign IDs corresponds to seasons: green, spring; blue, summer; purple, autumn.

**Table S1.** Summary of *Zingel asper* diet pooled across all sampling campaigns. Only prey items with a relative diet occurrence of ≥0.05 are included. Relative occurrence indicates the proportion of *Z. asper* diets that contained each prey taxa. Average abundance corresponds to total consumption [based on minimum number of individuals (MNI)] divided by the number of *Z. asper* individuals. Note that diet metrics for Heptageniidae does not include *Epeorus* or *Rhithrogena*, and Chrionomidae does not include Orthocladiinae.

**Table S2.** The composition of the macroinvertebrate community used for electivity tests. Each value indicates the relative abundance (%) of each taxon in the total prey community per sampling campaign. Only taxa that occurred in at least 5% of *Zingel asper* diets (pooled across all sampling campaigns) were included.

**Table S3.** Summary of *Baetis* mean density in the environment (Inv. m^2^) and in the diet of *Zingel asper* (diet). Values correspond to the average abundance (i.e., total consumption/number of *Z. asper* individuals) of each prey taxa in the *Z. asper* diet [based on minimum number of individuals (MNI)].

**Data S1.** Supporting information.
