# Supplementary figures and images for: A very picky eater: Species‐level prey selection in the endangered Rhone streber [ Zingel asper (L. 1758)]
Source: J Fish Biol. 2025 May 26;107(3):1060–6. doi: 10.1111/jfb.70083 (PMC12463753; doi:10.1111/jfb.70083)

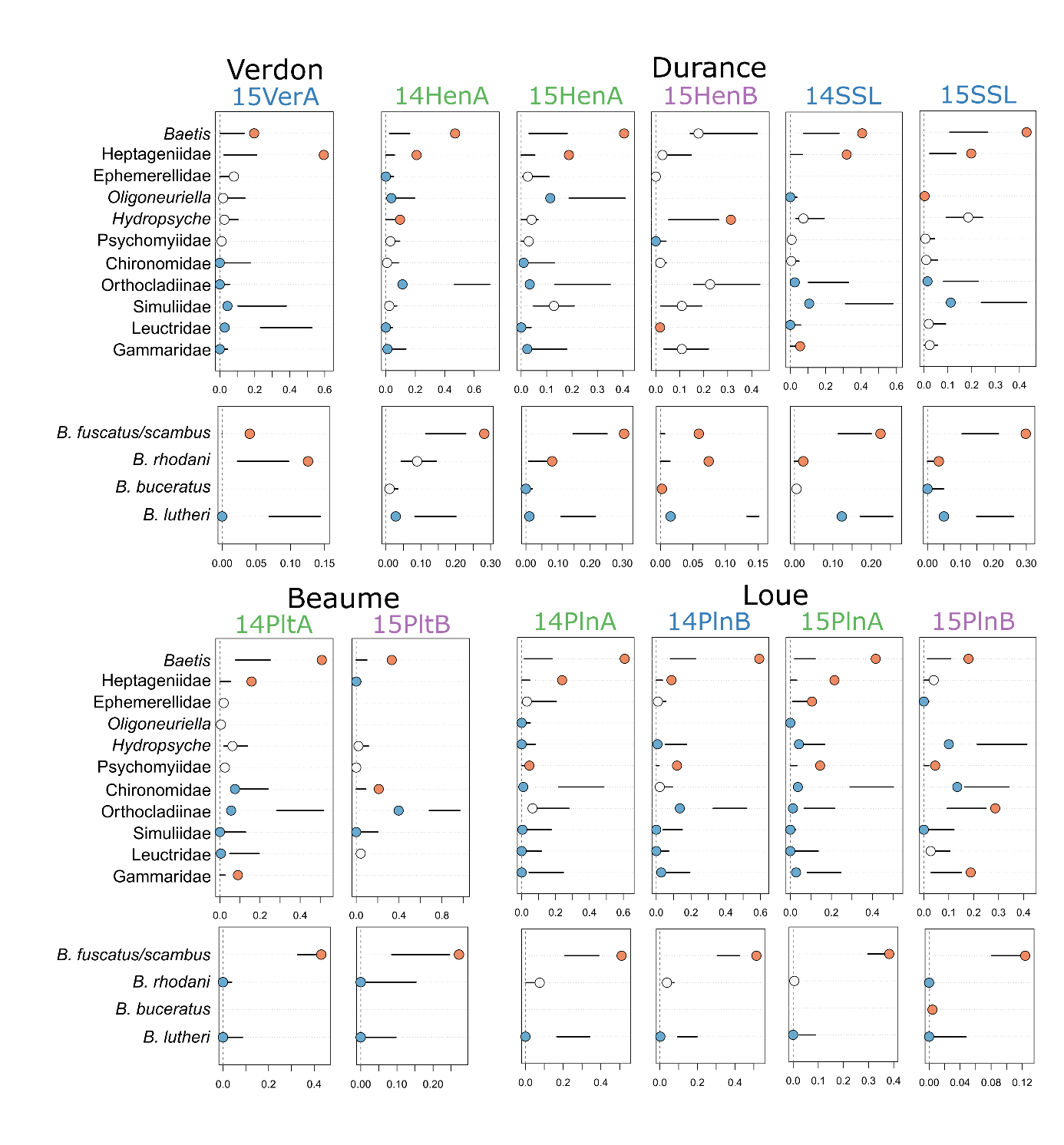

Supplement: Supplementary file 1 — Figure S1. Zingel asper prey preferences at genus‐ and family levels (above) and within the Baetis genus (below). The position of dots along the x‐axis indicates observed consumption (dietary proportions; 0–1); the colour of dots indicates deviation from expected frequencies of trophic interactions; blue, lower consumption than expected; white, as expected (in proportion to relative environmental abundance); red, higher than expected (consumed more frequently than expected). Horizontal lines denote 95% confidence limits of null model expectations of prey consumption. Genus‐ and family‐level tests of prey preferences were extracted from Villsen et al. (2024). Note that selection tests for Heptageniidae do not include Epeorus or Rhithrogena, and Chrionomidae does not include Orthocladiinae. The colour of campaign IDs corresponds to seasons: green, spring; blue, summer; purple, autumn. [file JFB-107-1060-s001.docx]
