# Supplementary material for: A very picky eater: Species‐level prey selection in the endangered Rhone streber [ Zingel asper (L. 1758)]
Source: J Fish Biol. 2025 May 26;107(3):1060–6. doi: 10.1111/jfb.70083 (PMC12463753; doi:10.1111/jfb.70083)
Supplement: Supplementary file 2 — Table S1. Summary of Zingel asper diet pooled across all sampling campaigns. Only prey items with a relative diet occurrence of ≥0.05 are included. Relative occurrence indicates the proportion of Z. asper diets that contained each prey taxa. Average abundance corresponds to total consumption [based on minimum number of individuals (MNI)] divided by the number of Z. asper individuals. Note that diet metrics for Heptageniidae does not include Epeorus or Rhithrogena, and Chrionomidae does not include Orthocladiinae. [file JFB-107-1060-s003.docx]

| **Prey** | **Relative occurrence** | **Average abundance** |
| --- | --- | --- |
| *Baetis fuscatus/scambus* | 0.77 | 1.56 |
| Heptageniidae | 0.56 | 0.78 |
| Orthocladiinae | 0.34 | 0.55 |
| *Hydropsyche* | 0.31 | 0.37 |
| Gammaridae | 0.26 | 0.30 |
| *Baetis rhodani* | 0.21 | 0.25 |
| *Baetis lutheri* | 0.18 | 0.23 |
| Simuliidae | 0.17 | 0.28 |
| Psychomyiidae | 0.15 | 0.17 |
| Chironomidae | 0.10 | 0.14 |
| Oligoneuriella | 0.08 | 0.09 |
| *Baetis buceratus* | 0.06 | 0.06 |
| Ephemerellidae | 0.05 | 0.05 |
| *Procloeon* | 0.05 | 0.06 |
