# Supplementary material for: A very picky eater: Species‐level prey selection in the endangered Rhone streber [ Zingel asper (L. 1758)]
Source: J Fish Biol. 2025 May 26;107(3):1060–6. doi: 10.1111/jfb.70083 (PMC12463753; doi:10.1111/jfb.70083)
Supplement: Supplementary file 3 — Table S2. The composition of the macroinvertebrate community used for electivity tests. Each value indicates the relative abundance (%) of each taxon in the total prey community per sampling campaign. Only taxa that occurred in at least 5% of Zingel asper diets (pooled across all sampling campaigns) were included. [file JFB-107-1060-s002.docx]

| **River** | **Campaign ID** | ***Baetis fuscatus/scambus*** | ***Baetis rhodani*** | ***Baetis buceratus*** | ***Baetis lutheri*** | **Heptageniidae** | ***Procloeon*** | **Ephemerellidae** | **Oligoneuriella** | ***Hydropsyche*** | **Limoniidae** | **Chironomidae**  (excluding Orthocladiinae) | **Simuliidae** | **Psychomyiidae** | **Orthocladiinae** | **Gammaridae** |
| --- | --- | --- | --- | --- | --- | --- | --- | --- | --- | --- | --- | --- | --- | --- | --- | --- |
| Durance | 14HenA | 3.28 | 1.76 | 0.19 | 2.74 | 1.63 | 0.00 | 1.37 | 11.54 | 2.12 | 0.43 | 3.38 | 2.56 | 3.72 | 58.60 | 6.66 |
|  | 15HenA | 4.98 | 0.82 | 0.10 | 3.99 | 1.38 | 0.13 | 4.62 | 30.03 | 2.11 | 0.08 | 6.13 | 12.36 | 0.50 | 23.32 | 9.46 |
|  | 15HenB | 0.13 | 0.28 | 0.03 | 27.06 | 6.65 | 0.00 | 0.01 | 0.00 | 15.04 | 0.51 | 0.90 | 9.71 | 0.51 | 28.55 | 10.61 |
|  | 14SSL | 7.13 | 0.08 | 0.80 | 9.14 | 2.15 | 0.00 | 0.00 | 0.71 | 10.32 | 0.15 | 1.16 | 46.06 | 0.08 | 20.69 | 1.51 |
|  | 15SSL | 7.76 | 0.12 | 0.13 | 10.80 | 7.57 | 0.00 | 0.00 | 0.00 | 16.74 | 0.37 | 2.16 | 35.46 | 1.42 | 15.34 | 2.12 |
| Verdon | 15VerA | 0.00 | 3.02 | 0.00 | 5.87 | 17.13 | 0.04 | 5.08 | 9.25 | 5.48 | 0.31 | 13.41 | 37.42 | 0.13 | 1.93 | 0.92 |
| Beaume | 14PltA | 5.89 | 0.02 | 0.00 | 0.99 | 0.74 | 0.48 | 5.21 | 0.28 | 10.53 | 2.29 | 41.71 | 0.61 | 0.89 | 13.91 | 16.46 |
|  | 15PltB | 4.49 | 0.01 | 0.00 | 1.08 | 1.25 | 0.29 | 0.29 | 0.00 | 32.84 | 0.73 | 25.71 | 6.90 | 0.32 | 17.16 | 8.94 |
| Loue | 14PlnA | 15.14 | 0.53 | 0.00 | 1.91 | 1.59 | 0.86 | 0.50 | 0.32 | 8.05 | 0.52 | 17.58 | 7.08 | 0.72 | 44.78 | 0.41 |
|  | 14PlnB | 1.25 | 0.53 | 0.00 | 0.22 | 0.45 | 0.12 | 0.00 | 0.00 | 3.38 | 0.14 | 1.92 | 6.92 | 0.02 | 85.05 | 0.00 |
|  | 15PlnA | 4.02 | 0.47 | 0.00 | 3.23 | 0.86 | 0.00 | 10.59 | 1.16 | 2.16 | 5.05 | 34.43 | 7.97 | 0.68 | 16.05 | 13.33 |
|  | 15PlnB | 10.07 | 1.16 | 0.00 | 3.97 | 1.08 | 0.06 | 2.26 | 0.00 | 10.88 | 1.40 | 4.65 | 9.10 | 0.17 | 43.14 | 12.07 |
