# Supplementary material for: A very picky eater: Species‐level prey selection in the endangered Rhone streber [ Zingel asper (L. 1758)]
Source: J Fish Biol. 2025 May 26;107(3):1060–6. doi: 10.1111/jfb.70083 (PMC12463753; doi:10.1111/jfb.70083)
Supplement: Supplementary file 4 — Table S3. Summary of Baetis mean density in the environment (Inv. m2) and in the diet of Zingel asper (diet). Values correspond to the average abundance (i.e., total consumption/number of Z. asper individuals) of each prey taxa in the Z. asper diet [based on minimum number of individuals (MNI)]. [file JFB-107-1060-s004.docx]

| **Site** | **Campaign ID** | **Year** | **Season** | ***B. fuscatus /scambus*** | ***B. fuscatus*** | ***B. scambus*** | ***B. rhodani*** | | ***B. buceratus*** | | ***B. lutheri*** | |
| --- | --- | --- | --- | --- | --- | --- | --- | --- | --- | --- | --- | --- |
|  |  |  |  | **Inv. m²** | **Diet** | **Diet** | **Inv. m²** | **Diet** | **Inv. m²** | **Diet** | **Inv. m²** | **Diet** |
| Durance (Hen) | 14HenA | 2014 | Spring | 132 | 1.18 | 0.03 | 70 | 0.49 | 8 | 0.06 | 110 | 0.12 |
|  | 15HenA | 2015 | Spring | 182 | 2.10 | 0.03 | 30 | 0.63 | 4 | - | 145 | 0.07 |
|  | 15HenB | 2015 | Autumn | 3 | 0.44 | - | 6 | 0.26 | 1 | 0.04 | 540 | 0.17 |
| Durance (SSL) | 14SSL | 2014 | Summer | 420 | 1.42 | - | 5 | 0.27 | 47 | - | 539 | 0.31 |
|  | 15SSL | 2015 | Summer | 157 | 1.16 | - | 2 | 0.16 | 3 | 0.05 | 219 | 0.71 |
| Verdon | 15VerA | 2015 | Summer | - | 0.20 | - | 22 | 0.50 | - | - | 42 | - |
| Loue | 14PltA | 2014 | Spring | 244 | 2.71 | 0.76 | 29 | 0.52 | - | - | 196 | - |
|  | 15PltB | 2014 | Summer | 329 | 2.84 | 0.82 | 38 | 0.29 | - | - | 130 | 0.02 |
|  | 14PlnA | 2015 | Summer | 254 | 2.34 | 0.66 | 1 | 0.02 | - | - | 43 | - |
|  | 14PlnB | 2015 | Autumn | 102 | 0.54 | - | 0 | - | - | 0.02 | 25 | - |
| Beaume | 15PlnA | 2014 | Spring | 630 | 2.37 | - | 22 | - | - | - | 79 | - |
|  | 15PlnB | 2015 | Autumn | 23 | 0.69 | - | 10 | - | - | - | 4 | - |
