# Supplementary material for: A very picky eater: Species‐level prey selection in the endangered Rhone streber [ Zingel asper (L. 1758)]
Source: J Fish Biol. 2025 May 26;107(3):1060–6. doi: 10.1111/jfb.70083 (PMC12463753; doi:10.1111/jfb.70083)
Supplement: Supplementary file 5 — Data S1. Supporting information. [file JFB-107-1060-s005.docx]

# Supplementary materials

***Description of faeces metabarcoding protocol***

*Zingel asper* faeces were directly collected from *Z. asper* individuals by pressing the abdomen by hand to drain out faeces. Faeces were immediately placed in a 2ml vial containing 96% ethanol and stored at -20°C prior to DNA extractions. Faecal DNA extractions were conducted in a room dedicated to the handling of degraded DNA (*Plateforme ADN Dégradé* of the LabEx CeMEB, Montpellier, France) following the method described by Corse et al., (2017) and the specific safety measures described by (Monti et al., (2015). The DNA was extracted from the whole faeces for all individuals. In order to further minimize cross-sample contamination, extraction series were limited to 25 samples, including 21 Z. asper faeces, two ‘alien’ faeces (i.e. from marine or non-European continental predators; see: Corse et al., 2019), one negative control for extraction and one negative control for aerosols (for more details on controls, see: Corse et al., 2017, 2019). Additionally, the analyses also included two distinct mock samples communities (described in Corse et al. 2019) as both positive controls and standards across MiSeq runs. Samples and controls were amplified by PCR in triplicates using three primer sets (MFZR, ZFZR and LFCR; Corse et al. 2019) that target ~150bp overlapping sequences located in the 5’ end of the Cytochrome c oxidase subunit I gene (COI). Thus, nine separate PCRs were generated per sample/control. The PCR-enrichment step included the one-locus-several-primers (OLSP) strategy developed by Corse et al., (2019), which aims to minimize false negatives in metabarcoding data by using several primer sets that target overlapping but complementary invertebrate taxa. Amplicons were then processed and sequenced on an Illumina MiSeq v3 platform (as detailed in Corse et al. 2017).

| **Primer set** | **Primer name** | **Forward (F) / Reverse(R)** | **Sequence (5'-3')** | **Reference** |
| --- | --- | --- | --- | --- |
|  |  |  |  |  |
| MFZR | Uni-Minibar-F1 | F | TCCACTAATCACAARGATATTGGTAC | Meusnier et al. 2008 |
|  | ZBJ-ArtR2c | R | WACTAATCAATTWCCAAATCCTCC | Zeale et al. 2011 |
| ZFZR | ZBJ-ArtF1c | F | AGATATTGGAACWTTATATTTTATTTTTGG | Zeale et al. 2011 |
|  | ZBJ-ArtR2c | R | WACTAATCAATTWCCAAATCCTCC | Zeale et al. 2011 |
| LFCR | LepLCO | F | RKTCAACMAATCATAAAGATATTGG | Corse et al. 2019 |
|  | McoiR2 | R | CCBCCRATTAWAATKGGTATHAC | Corse et al. 2019 |

High-throughput sequencing (HTS) data were then filtered using the ASV-based (Amplicon Sequence Variant) procedure developed by (Corse et al., 2017) which is now implemented in VTAM (Validation and Taxonomic Assignment of Metabarcoding data; González et al., 2023). In short, this procedure explicitly uses the sequencing outputs of negative and positive controls, and exogenous samples in order to minimise false positives in faecal samples (i.e. experimental/molecular artefacts such as PCR/sequencing errors, tag switching and cross-sample contaminations). Reproducibility of ASVs was further ensured by explicitly using the sequencing outputs of technical (PCR) replicates. We also discarded chimeras and pseudogenes. Finally, the ASVs obtained from the different primer sets that were identical in their overlapping regions (~130bp) were combined into contigs (further details in: Corse et al., 2017, 2019).

The taxonomic assignment of ASVs/contigs was conducted as detailed in (Corse et al., 2017). As *Z. asper* mainly feed on macroinvertebrates but can also fed on fishes (Cavalli et al., 2003; Corse et al., 2019), we considered macroinvertebrates and fishes as relevant prey and collectively referred to them as Macrometazoans. All other taxa (listed in Corse et al., 2019) were excluded from the analyses. Prey abundance in each diet was estimated using the Minimal Number of Individuals statistic (MNI; White, 1953). MNI is a quantitative statistic that corresponds to the number of distinct ASVs/contigs validated in each sample for a given prey taxon (see Corse et al., 2017).

***Description of macroinvertebrate sampling protocol***

To obtain a fine-scale estimation of the ecological opportunities of Z. asper in each sampling campaign we performed extensive prey community and habitat sampling. For each sampling campaign, we collected between 45 to 90 prey community and habitat samples. Prey community sampling was performed 1-2 days before or after fish sampling. The sampling effort was distributed among the representative habitats (i.e., rifﬂes, runs, glides and rare pools) in the fishing area. Samples were collected using a Surber sampler by perpendicular transects between riverbanks, from downstream to upstream. One to ﬁve Surber samples (0.05 m2) were collected per transect in all accessible habitats (i.e., < 80 cm depth; <2 m.s−1 water velocity). Macroinvertebrates were immediately stored in 96% ethanol for subsequent identification in the laboratory. Macroinvertebrates were assigned to genus or species using morphological criteria (Tachet et al., 2010). When this was not feasible using morphology however (e.g., due to the development stage of larvae), taxa were aggregated at higher taxonomic levels (i.e., family or subfamily).

## References :

Corse, E., Meglécz, E., Archambaud, G., Ardisson, M., Martin, J.-F., Tougard, C., Chappaz, R., & Dubut, V. (2017). A from-benchtop-to-desktop workflow for validating HTS data and for taxonomic identification in diet metabarcoding studies. *Molecular Ecology Resources*, 17(6), e146–e159. https://doi.org/10.1111/1755-0998.12703

Corse, E., Tougard, C., Archambaud‐Suard, G., Agnèse, J. F., Messu Mandeng, F. D., Bilong Bilong, C. F., ... & Dubut, V. (2019). One‐locus‐several‐primers: A strategy to improve the taxonomic and haplotypic coverage in diet metabarcoding studies. *Ecology and Evolution*, 9(8), 4603-4620.

González, A., Dubut, V., Corse, E., Mekdad, R., Dechatre, T., Castet, U., Hebert, R., & Meglécz, E. (2023). VTAM: A robust pipeline for validating metabarcoding data using controls. *Computational and Structural Biotechnology Journal*, *21*, 1151–1156. https://doi.org/10.1016/j.csbj.2023.01.034

Meusnier, I., Singer, G. A., Landry, J. F., Hickey, D. A., Hebert, P. D., & Hajibabaei, M. (2008). A universal DNA mini-barcode for biodiversity analysis. *BMC Genomics*, *9*, 214. https://doi.org/10.1186/1471-2164-9-214

Monti, F., Duriez, O., Arnal, V., Dominici, J. M., Sforzi, A., Fusani, L., Grémillet, D., & Montgelard, C. (2015). Being cosmopolitan: Evolutionary history and phylogeography of a specialized raptor, the Osprey *Pandion haliaetus*. *BMC Evolutionary Biology*, *15(1)*, 255. https://doi.org/10.1186/s12862-015-0535-6

Tachet, H., Richoux, P., Bournaud, M., & Usseglio-Polatera, P. (2010). *Invertébrés d’eau douce: Systématique, biologie, écologie*. Paris: CNRS éditions.

White, T. E. (1953). A method of calculating the dietary percentage of various food animals utilized by Aboriginal peoples. *American Antiquity*, *18(4)*, 396–398. https://doi.org/10.2307/277116

Zeale, M. R. K., Butlin, R. K., Barker, G. L. A., Lees, D. C., & Jones, G. (2011). Taxon-specific PCR for DNA barcoding arthropod prey in bat faeces. *Molecular Ecology Resources*, 11(2), 236–244. https://doi.org/10.1111/j.1755-0998.2010.02920.x
